# The complexities of insulin allergy: a case and approach

**DOI:** 10.1186/s13223-021-00554-1

**Published:** 2021-07-29

**Authors:** Babak Aberumand, Samira Jeimy

**Affiliations:** 1 Division of Allergy and Immunology, Department of Medicine, University of Toronto, 30 Bond St. M5B 1W8, Toronto, ON Canada; 2 Division of Allergy and Immunology, Department of Medicine, Western University, London, ON Canada

**Keywords:** Insulin allergy, Anaphylaxis, Diabetes mellitus, Type I hypersensitivity

## Abstract

**Background:**

Insulin hypersensitivity is rare, but challenging for individuals with diabetes. The prevalence of insulin allergy has decreased since the introduction of human recombinant insulin preparations. Hypersensitivity reactions range from injection site erythema and swelling, to anaphylaxis. While some reactions are to excipients (zinc, protamine, metacresol), many are to recombinant insulin itself. We present a case of type 1 hypersensitivity to various preparations of insulin in a patient with insulin-dependent type 2 diabetes mellitus (T2DM).

**Case presentation:**

A 61-year-old woman with a 30-year history of insulin-dependent T2DM was referred for evaluation of reactions to insulin. She had two episodes over 5-months; both required Emergency Department visits and epinephrine administration. The first episode entailed a burning sensation of the extremities and nausea, immediately after injecting NovoRapid^®^ insulin. The second event entailed a similar reaction but this time there was also angioedema of the upper airway with difficulty breathing and hypotension, immediately after injecting Levemir^®^ and NovoRapid^®^, and taking metformin. There were no cofactors such as exercise, infectious illness, or NSAIDs use. Skin testing was performed with metformin, Lantus^®^, Humalog^®^, NovoRapid^®^, glulisine, insulin regular, NPH, Levemir^®^ and the excipient protamine, as per published testing concentrations. Metacresol was not tested as its use was restricted by the hospital pharmacy. Insulin preparations with and without metacresol were included in testing however. A clinic staff served as a negative control. The patent had negative testing with protamine, but sensitization to all insulin preparations. Metformin skin testing and challenge along with latex IgE were negative. Subsequently, she underwent intentional weight loss of 70 lb, and was started on oral hypoglycemics with good effect.

**Conclusions:**

Our case highlights the importance of diagnosing insulin allergy through a detailed history and focused testing. Therapeutic strategies include avoidance and insulin alternatives, alternate insulin preparations, or desensitization. In severe recurrent hypersensitivity reactions, Omalizumab or pancreatic transplantation have been effective.

## Introduction

Insulin is a polypeptide hormone produced by beta cells of the pancreatic islets in response to hyperglycemia, and is heavily involved in the regulation of serum glucose levels [1]. It is used as the mainstay of treatment in type 1 diabetes mellitus (T1DM) and severe T2DM patients with poor glycemic control despite maximal oral hypoglycemics therapy [2, 3]. Hypersensitivity reactions to insulin are rare but Type I, Type III and Type IV hypersensitivity reactions have been described. IgE-mediated reactions classically occurring within 1 hour of injection [4, 5]. Signs and symptoms range from localized erythema and swelling at the site of injection to severe generalized reactions such as anaphylaxis [6, 7]. Hypersensitivity reactions to insulin were significantly more prevalent in animal preparations of insulin such as bovine and porcine insulins. However, with the introduction of human recombinant insulin, and minor modifications from the amino acid sequence of human insulin, the prevalence of insulin hypersensitivity reactions has significantly decreased and is estimated at less than 2.4% in patients on insulin therapy [8].

Despite recombinant human insulin being the culprit in some of the cases of insulin allergy [9], patients may also react to the inactive excipients such as protamine, zinc or metacresol in the insulin preparations [8, 10, 11]. Therapeutic options in patients with insulin allergy can thus be challenging. In the present study, we describe a case of a woman with T2DM on insulin for over 30 years who experienced two immediate reactions following the administration of NovoRapid^®^ insulin and subsequently Levemir^®^ and NovoRapid^®^ insulin. We provide an approach to evaluating patients with a hypersensitivity reaction to insulin and an overview of the management options currently available.

## Case presentation

A 61-year-old woman was referred to the Allergy Clinic at Western University for evaluation of an IgE-mediated reaction to insulin. She had two episodes over a 5-month period that required a visit to the Emergency Department (ED), requiring treatment with epinephrine to achieve resolution of her symptoms. The first event she experienced entailed a burning sensation in her hands and feet with nausea immediately after injecting NovoRapid^®^ insulin subcutaneously. The second event caused a similar reaction but there was also throat swelling and difficult breathing immediately after taking metformin, Levemir^®^ and NovoRapid^®^. There was no rash, vomiting, diarrhea or loss of consciousness. In the ED, she was found to be hypotensive. There were no cofactors such as exercise or other medications including NSAIDs taken at the time of these reactions. On both occasions, the insulin used were from a fresh vial stored in her refrigerator. She did not have any previous reactions to insulin and had been on the same medication for over 30 years without missing any doses. She had a medical history comprising of longstanding T2DM with a recent hemoglobin A1c level of 8.9%, Lyme disease in 2018 and herpes zoster in 2019. There was no personal history of previous allergies to drugs or insect stings, atopy or autoimmune disease. Her medications included zopiclone 5 mg once daily, detemir insulin 20 units every morning and Novorapid^®^ insulin 10 units twice daily. Both forms of insulin were held after the reactions mentioned above.

In clinic, she underwent skin-prick testing (SPT) to metformin, Lantus^®^, Humalog^®^, NovoRapid^®^, Apidra^®^, Insulin Regular, NPH, Detemir, and the additive protamine. NPH was diluted 30-fold to 333 μg/mL for testing, to approximate concentration in undiluted NPH insulin (350 μg/mL). Metacresol testing was not performed as its use was restricted by the hospital pharmacy but she had positive wheal and flare responses to non–metacresol–containing insulin preparations. Insulin preparations containing zinc and without zinc were included in testing. A positive skin-prick test was defined as a wheal size of greater than 3 mm than the negative control that developed within 15 min. All skin-prick test results were interpreted by an Allergist and Clinical Immunologist and validated with an appropriate histamine (10 mg/mL) positive control and saline negative control.

**Fig. 1:**
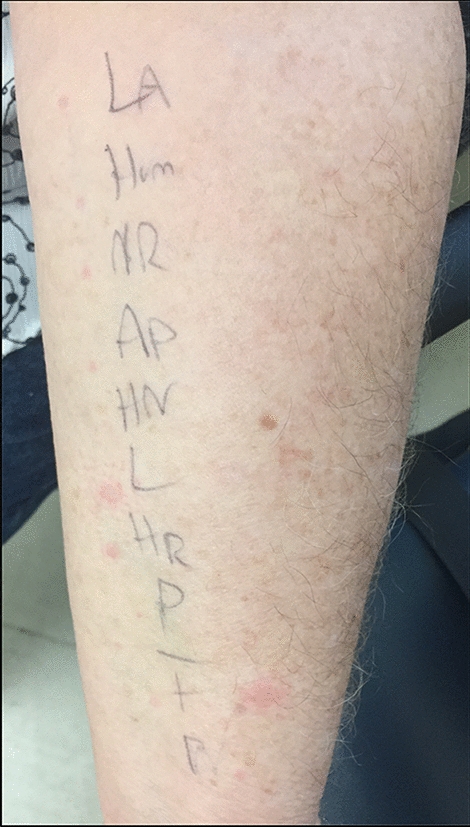
Skin-prick testing to various insulin preparations and protamine. Insulin preparations: *LA* lantus; *Hum* humalog; *NR* novorapid; *AP* apidra (glulisine); HN—NPH; L—detemir (levemir); HR—insulin regular; non-insulin preparations: *P* protamine

**Fig. 2:**
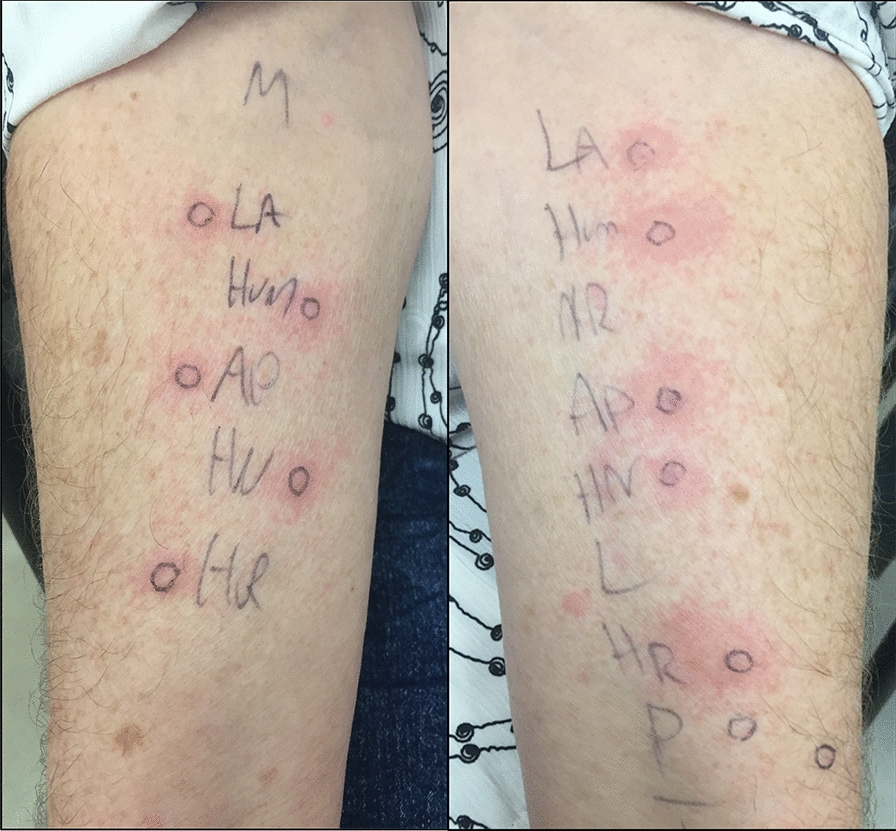
Intradermal testing at 1:10 (left) and 1:100 dilutions (right) with the various insulin preparations and diluted protamine. Insulin preparations: *LA* lantus; *Hum* humalog; *NR* novorapid; *AP* apidra (glulisine); HN—NPH; L—detemir (levemir); HR—insulin regular; non-insulin preparations: *M* meformin; *P* protamine

Positive SPT to the long-acting basal insulin Detemir was noted but negative to metformin, the other insulin formulations such as Lantus^®^, NPH, Humalog^®^, Novorapid^®^, Apidra^®^, Insulin Regular, and the additive protamine (Fig. 1). Subsequently, intradermal testing at 1:10 and 1:100 dilutions with the various insulin preparations along with protamine at 3 mcg/mL and 0.3 mcg/m indicated sensitization to all insulin preparations at both concentrations (Fig. 2). Protamine at both concentrations was negative. A positive intradermal skin test was defined as a wheal size of greater than 3 mm than the negative control that developed at least 15 min after the allergen was pricked to the skin of the participant’s forearm. Again, the results of the intradermal skin testing were validated with an appropriate histamine (10 mg/mL) positive control and saline negative control. Baseline and intermittent clinical exams, vital sign assessment, and blood glucose measurements were performed during the SPT and intradermal skin testing with no changes to the clinical exam. Blood glucose levels remained stable ranging from 9 to 12 mmol/L. All tests were applied to a healthy volunteer for a negative control. Serum IgE insulin was elevated at 2.85 kU/L. Serum tryptase and serum C4 levels were normal at 3.9 ng/mL and 0.34 g/L respectively. Latex IgE level was also normal at  < 0.1 kU/L. Oral challenge to metformin was negative.

She was diagnosed with an IgE-mediated anaphylactic reaction to insulin and advised to avoid insulin, unless she underwent desensitization therapy in a monitored setting followed by continuous use of an insulin pump. Two epinephrine autoinjectors were provided and recommended to be on hand at all times after a discussion and shared decision making with the patient.

## Discussion and conclusion

Since the introduction of the first recombinant human insulin, Humulin, the prevalence of insulin use has increased vastly [12], and various forms have been developed ranging from short-acting to long-acting (Table 1). Animal insulin formulations were significantly immunogenic, with bovine insulin being more likely to result in a reaction than porcine insulin [1, 13]. However, since the introduction of recombinant human insulin, the immunogenicity has drastically decreased [5, 14]. Despite this, hypersensitivity reactions can still occur to various components of an insulin preparation including additives and preservatives such as zinc, protamine and metacresol, and also the insulin component as well [8–11].

**Table 1:** Types of recombinant insulin with their respective clinically relevant non-medicinal ingredients

Recombinant insulin type (name and brand)	Clinically relevant non-medicinal ingredients
Bolus	
Rapid-acting insulin analogues	
Insulin aspart (NovoRapid^®^)	Metacresol
	Zinc chloride solution
	Disodium phosphate dihydrate
	Glycerol
	Hydrochloric acid
	Sodium chloride solution
Insulin faster aspart (Fiasp^®^)	Metacresol
	Zinc acetate
	Phenol
	Disodium phosphate dihydrate
	Glycerol
	Hydrochloric acid
	Sodium chloride solution
	L-arginine HCl
	Niacinamide (vitamin B_3_)
Insulin lispro (Humalog^®^, Humalog 100 units/mL)	Metacresol
	Zinc (as ion)
	Dibasic sodium phosphate
	Glycerol
	Hydrochloric acid
	Sodium hydroxide
Insulin lispro (Humalog^®^, Humalog 200 units/mL)	Metacresol
	Zinc oxide
	Sodium hydroxide
	Glycerol
	Hydrochloric acid
	Tromethamine
Insulin lispro (Admelog^®^)	Metacresol
	Zinc oxide
	Dibasic sodium phosphate
	Glycerol
	Hydrochloric acid
	Sodium hydroxide
Insulin glulisine (Apidra^®^)	Metacresol
	Sodium chloride
	Polysorbate 20
	Hydrochloric acid
	Tromethamine
Short-acting Insulins	
Insulin regular (Humulin R^®^)	Metacresol
	Sodium hydroxide
	Glycerol
	Hydrochloric acid
Novolin^®^ge Toronto	Metacresol
	Zinc chloride
	Glycerol
	Sodium hydroxide
	Hydrochloric acid
Intermediate-acting insulins	
Humulin N^®^	Protamine sulfate
	Zinc oxide
	Dibasic sodium phosphate
	Phenol
Novolin^®^ge NPH	Zinc chloride
	Sodium hydroxide
	Phenol
	Disodium phosphate dihydrate
Basal	
Long-acting basal insulin analogues	
Insulin detemir (Levemir^®^)	Metacresol
	Zinc acetate
	Glycerol
	Sodium hydroxide
	Phenol
	Disodium phosphate dihydrate
	Hydrochloric acid
Insulin glargine 100 units/mL (Lantus^®^)	Metacresol
	Zinc
	Glycerol 85%
	Polysorbate 20
	Hydrochloric acid
	Sodium hydroxide
Insulin glargine 100 units/mL (Basaglar^®^)	Metacresol
	Zinc oxide
	Glycerol 85%
	Polysorbate 20
	Hydrochloric acid
	Sodium hydroxide
Insulin glargine 300 units/mL (Toujeo^®^)	Metacresol
	Zinc chloride
	Glycerol 85%
	Hydrochloric acid
	Sodium hydroxide
Insulin degludec (Tresiba^®^)	Metacresol
	Zinc acetate
	Glycerol
	Phenol
Pre-mixed insulins	
Premixed regular insulin	
Humulin 30/70^®^	Protamine sulfate
	Zinc oxide
	Dibasic sodium phosphate
	Phenol
Novolin^®^ge 30/70 Novolin^®^ge 40/60 Novolin^®^ge 50/50	Metacresol
	Protamine sulphate
	Zinc chloride
	Disodium phosphate dihydrate
	Glycerol
	Phenol
	Hydrochloric acid
	Sodium hydroxide
Premixed insulin analogues	
Biphasic insulin aspart (NovoMix 30^®^)	Protamine sulfate
	Metacresol
	Zinc chloride
	Glycerol
	Phenol
	Sodium chloride
	Dibasic sodium phosphate dihydrate
	Sodium hydroxide,
	Hydrochloric acid
Insulin lispro/lispro protamine (Humalog Mix 25^®^, Humalog Mix 50^®^)	Protamine sulfate
	Zinc oxide
	Phenol

### Clinical presentation

Type I immediate hypersensitivity reactions are the most common type of allergic reaction to recombinant human insulin [5]. Typically, the reaction occurs 1 week after initiation of insulin therapy and typically within 1 h of administration. Biphasic reactions have occurred 4–6 h after the initial reaction [5, 7]. Patients on insulin therapy, who have interruptions in therapy ranging from months to years, have reported a Type I immediate hypersensitivity reaction after resuming insulin [7]. Type III [14] and Type IV reactions have also been described [5]. It is worthy to note that IgG antibodies are also involved in insulin resistance. Type IV hypersensitivity reactions tend to occur within 8–12 h of insulin use, peak at 24 h and last anywhere from 4 to 7 days [5, 7]. A case of a delayed reaction causing leukocytoclastic vasculitis in a patient with T1DM after recombinant insulin use has been reported [15]. Although extremely rare, local and systemic allergic reactions to endogenously secreted insulin in association with recombinant insulin therapy can occur [16].

### Pathogenesis

The pathogenesis of hypersensitivity reactions secondary to insulin is not well understood. It has been proposed that insulin molecules congregate into larger molecules which facilitate the formation of anti-insulin antibodies in subcutaneous tissue [17–19]. Newer recombinant insulins have less antigenicity as they are more rapidly absorbed, and hence are unlikely to form these aggregates, resulting in less mast cell exposure and the potential for the development of anti-insulin antibodies [20, 21]. The route of administration has also been suggested to play a role, with subcutaneous injections of insulin more likely to cause hypersensitivity as compared to intravenous injections [22]. Furthermore, genetics also seem to play a role as the HLA gene DR4 has been associated with high levels of insulin antibody formation [7].

### Work-up

Workup of insulin allergy involves a thorough history and physical exam to exclude other potential culprits. It is worth noting that contamination of the insulin needle can occur with latex when insulin is being drawn up, as latex is commonly found in the insulin vial [23]. As a result, latex allergy should be excluded, or latex-free insulin vials should be used if suspicion is high for a latex allergy. Allergy to excipients zinc, protamine and metacresol used with different formulations of insulin have been described as a potential cause of systemic reactions, and should be evaluated with skin prick testing (SPT) [9–12]. However, intradermal skin testing can also be used to diagnose insulin allergy and has been suggested to be more sensitive than SPT to detect insulin allergy [7]. As in this case, if commercial skin test kits are not available, insulin and protamine can be diluted for use for SPT and intradermal testing as per published dilution recommendations outlined in Table 2 [24]. Skin test results should be interpreted in association with the clinical symptoms as false positives do occur and have been noted in 28% of diabetic patients with low specific IgE titers [10]. In fact, it has been postulated that as high as 40% of asymptomatic diabetic patients may have a positive skin test result or specific IgE to insulin [25]. The presence of specific IgE insulin is more supportive of a type 1 hypersensitivity reaction and its absence less likely so. Circulating IgE levels can be elevated or absent in patients with insulin hypersensitivity reactions and may be low in acute reactions due to consumption [26].

**Table 2:** Proposed skin testing concentrations

Component	Recommended dilution	
	SPT	Intradermal
Insulin	No dilution	1/100000–1/10 dilution, then 1/1
Expedient (protamine, metacresol, zinc)	Vary depending on concentrations in insulin solutions. Attempt to dilute according to these concentrations	1/1000–1/10 dilution

### Management

The initial management of insulin anaphylaxis is similar to other forms of anaphylaxis; prompt administration of epinephrine is warranted. Cutaneous manifestations can be improved with the administration of second generation antihistamines. The use of systemic steroids can be considered, but special attention should be taken to monitor for hyperglycemia and insulin resistance. This can potentially increase exposure to the allergen as greater doses of insulin will be required. As well, the use of systemic steroids is not without risk of adverse side effects. Furthermore, evidence for corticosteroid or antihistamines to prevent a biphasic anaphylactic reaction is lacking [26, 27].

Long-term management of insulin allergy can be challenging and should involve a multidisciplinary team [28]. The mainstay of long-term management of insulin allergy is avoidance of insulin if possible or switching to another insulin preparation. In T2DM with confirmed insulin allergy, insulin should be switched to oral hypoglycemic agents if good glycemic control can be obtained [26]**.** Fortunately, this was successfully done with our patient as SPT and intradermal testing to other insulins was positive. Patients with confirmed sensitization to additives of commercial insulin preparations such as zinc, protamine or metacresol should be switched to a preparation that does not contain these excipients [9]. Unfortunately, in insulin-dependent conditions such as T1DM or acquired diabetics from a total pancreatectomy, insulin therapy is essential and discontinuing insulin is not feasible. As a result, switching to another insulin preparation that differs from the one that causes the insulin hypersensitivity reaction should be sought. Alternatively, insulin desensitization can be pursued, especially for those who are allergic to multiple forms of exogenous insulin. Tolerance is obtained through continuous or regular administration of incremental increasing doses of insulin analogues that typically have less antigenicity [3, 9, 20]. Longer acting insulin formulations for desensitization such as glargine have also been shown effective given its amino acid structure is less immunogenic and may actually inhibit immune reactions. Furthermore, glargine forms a slow dissolving precipitate after injection analogous to the antigen presentation with CSII [29]. Regular administration can be performed via intradermal, intravenous or subcutaneous injections [5, 9, 22]. The mechanism is believed to be centered around combination of decreased allergic mediators from mast cells through gradual stimulation of T-regulatory cells with incremental doses and the production of anti-insulin IgG-blocking antibodies [6, 9, 29]. The dose is increased in a graded fashion and has been shown to decrease symptoms and also IgE levels [9]. It is important to note that allergic symptoms can still persist despite the normalization of insulin-specific IgE and IgG levels [22]. Should a local allergic reaction occur, it is recommended that the dose at which the reaction occurred be repeated until there is resolution before increasing it. As opposed to a systemic reaction, where it is recommended that the dose be reduced to half of which caused the reaction [9]. Small doses of corticosteroids in the form of methylprednisolone 0.02 mg/1 U insulin or hydrocortisone 10 mg reduced to 2 mg after 2 weeks of use, mixed with insulin have also been shown to be effective in reducing local allergic reactions. The blood sugar level was controlled and there was a potential for gradual discontinuation of the corticosteroids after a few months of use [30, 31].

During desensitization, blood sugar levels need to be closely monitored and any significant increases should be managed with an insulin pump with insulin preparations that differ from the one used for desensitization. In the event that high insulin doses are used, a glucose solution should be administered to offset the effects of high levels of insulin [9]. Although uncommon, it is worthy to note that the lasting effects of desensitization can be minimal and short-term with a potential of returning symptoms [32]. Furthermore, there is a possibility of developing insulin resistance through the development of IgG antibodies [33]. Several protocols have been created to help ease the process of insulin desensitization in an inpatient and outpatient setting [3, 24] (Tables 3, 4).

**Table 3:** Desensitization protocol by CSII with a short-acting insulin. Adapted from Yuan et al. [3]

Day	Dose	Rate increase^a^	Target rate at the end of the day
1	0.01 IU/h of dilute insulin^b^	0.01 IU/h	0.24 IU/h
2	0.25 IU/h of non-dilute insulin	0.05 IU/h	1.4 IU/h
3	1.0 IU/h^c^	Add 4–6 IU to the bolus insulin infusion before each meal with the insulin pump	
4	Give the bolus insulin by subcutaneous injection using a syringe, with a CSII basal insulin infusion administered at the same time		
	Stop using CSII and gave the patient insulin therapy by alternative multiple subcutaneous injections		

^a^Check blood glucose levels and vitals q1h

^b^Short-action insulin preparation was diluted 10 times (from U100 to U10 by saline)

^c^Alternatively can decrease to 0.5 IU/h at night to avoid hypoglycemia in older patients

**Table 4:** Rapid desensitization protocol via subcutaneous insulin Glargine. Adapted from Hasani-Ranjbar et al. [24]

Day	Dose	Time between doses	Recommendations
1	Administer 0.0001U, 0.001U, 0.01U, 0.1U, 1U	30-min intervals	Check blood glucose levels and vitals q30 min
2	Administer 2U, 4U, 8U and 20U	30-min intervals	In case of a local reaction, treat with an antihistamine and the last dose is repeated until no reaction occurs and then the dose increases are continued
			If there are systemic reactions, treat with epinephrine and an antihistamine. Reduce the dose to one half
			At high insulin doses, a 10% glucose solution can be given to counteract the glucose-lowering effect

Continuous subcutaneous insulin infusion (CSII), first introduced in 1987, has also been shown to be effective and is typically implemented with a very low initial basal rate (i.e., 0.01 U/h) and increased slowly as tolerated. Rapid-acting analogs such as insulin lispro and insulin aspart are typically used as they are shorter-acting which reduces their exposure to mast cells and allows for quicker absorption and degradation at the injection site leading to reduced immunologic effects [3, 4, 29, 34–36]. CSII has been proposed to be as effective as the traditional regimen of desensitization [3, 37]. Concurrent intravenous insulin infusions should also be considered during the CSII to prevent diabetic ketoacidosis [38].

**Fig. 3:**
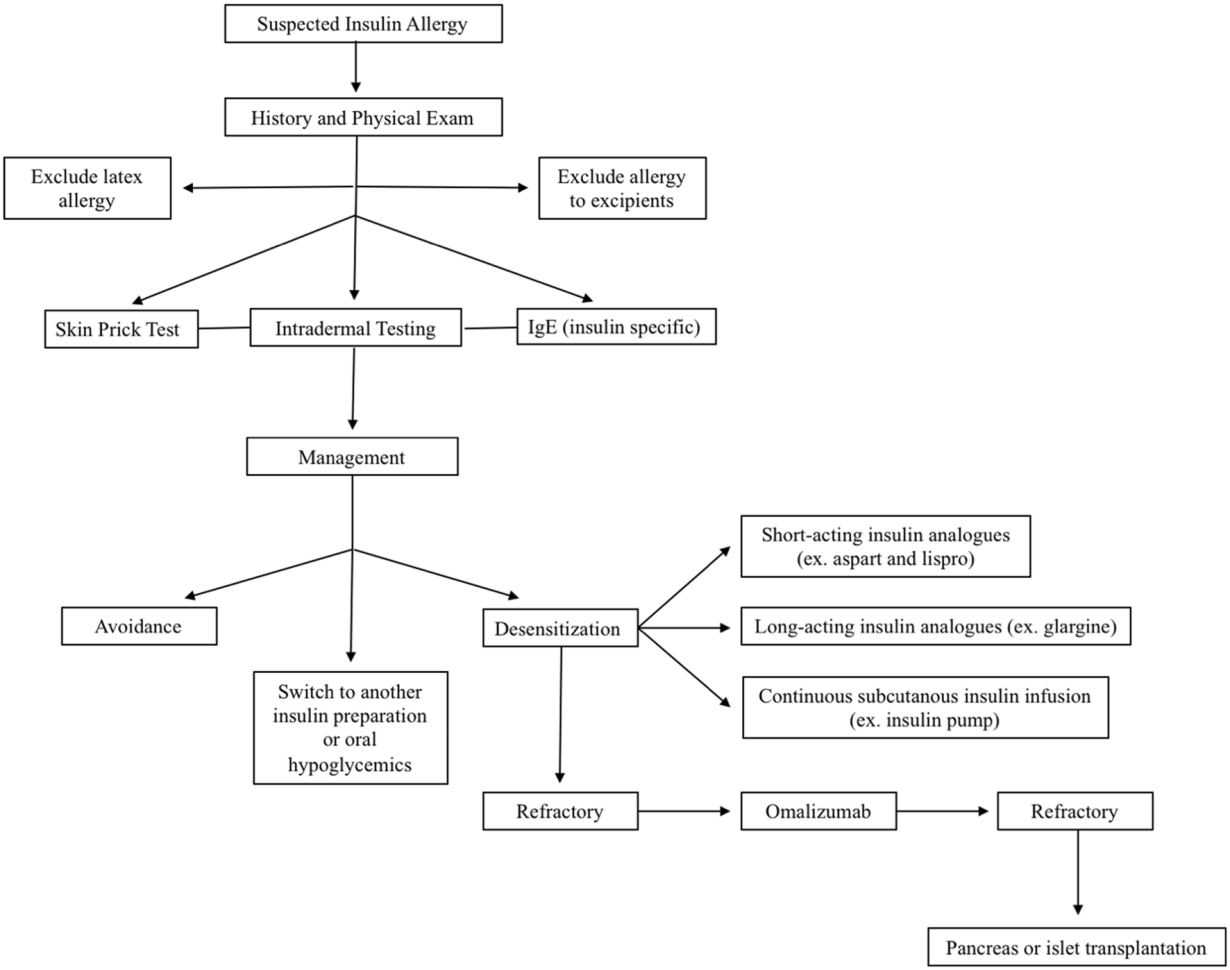
Algorithm for the workup and management of insulin allergy

Despite desensitization, recurrence of insulin allergy can occur [32]. In these situations, treatment with Omalizumab (Xolair^®^), an anti-IgE monoclonal antibody, has been successful in treating insulin allergy [39, 40]. In fact, the use of two targeted biologics in a two-step approach has similarly been successful in alleviating severe insulin allergy. Initially Rituximab, a B-cell depleting monoclonal antibody, was given as the patient had levels of IgE too high to meet criteria for Omalizumab administration. Once the IgE levels were reduced with Rituximab, Omalizumab was administered [41]. In more severe cases of insulin allergy, refractory to the above management options, pancreas transplant or islet transplantation can be considered. However, this is a last resort given the invasiveness of these procedures and the long-term consequences [42, 43]. An algorithm to the workup and management of insulin allergy is outlined in Fig. 3.

In conclusion, although rare, our case illustrates the potential complexities of the management of insulin allergy. Fundamental to making an accurate insulin allergy diagnosis, is a thorough history and focused physical exam. Particular attention should be placed on the timing of symptoms after insulin administration with suspicion heightening with smaller gaps. Investigations should focus on ruling other potential explanations for the symptoms including latex allergy and allergy to excipients used in insulin preparations. SPT and intradermal testing to both short-acting and long-acting insulins should be conducted along with IgE levels specific to insulin. Treatment strategies include insulin avoidance, the use of oral hypoglycemics, the use of alternate insulin preparations and/or desensitization. In recurrent insulin hypersensitivity reactions after prior desensitization, treatment with Omalizumab and. depending on severity, pancreatic or islet transplantation should be explored.

## Data Availability

The authors will consider making the relevant anonymized patient level data and material available on reasonable request.
